# Ten recommendations for hosting a Diversity, Equity, Inclusion, and Justice (DEIJ) journal club

**DOI:** 10.1371/journal.pcbi.1012166

**Published:** 2024-06-06

**Authors:** Roberto Efraín Díaz, Stephanie A. Wankowicz

**Affiliations:** 1 Helen Diller Family Comprehensive Cancer Center Office of Diversity, Equity, Inclusion, and Accessibility, University of California, San Francisco, San Francisco, California, United States of America; 2 Department of Bioengineering and Therapeutic Sciences, University of California, San Francisco, San Francisco, California, United States of America; Carnegie Mellon University, UNITED STATES

## Abstract

Despite advances and social progress, the exclusion of diverse groups in academia, especially science, technology, engineering, and mathematics (STEM) fields, across the US and Europe persists, resulting in the underrepresentation of diverse people in higher education. There is extensive literature about theory, observation, and evidence-based practices that can help create a more equitable, inclusive, and diverse learning environment. In this article, we propose the implementation of a Diversity, Equity, Inclusion, and Justice (DEIJ) journal club as a strategic initiative to foster education and promote action towards making academia a more equitable institution. By creating a space for people to engage with DEIJ theories* and strategize ways to improve their learning environment, we hope to normalize the practice and importance of analyzing academia through an equity lens. Guided by restorative justice principles, we offer 10 recommendations for fostering community cohesion through education and mutual understanding. This approach underscores the importance of appropriate action and self-education in the journey toward a more diverse, equitable, inclusive, and just academic environment.

*Authors’ note: We understand that “DEIJ” is a multidisciplinary organizational framework that relies on numerous fields of study, including history, sociology, philosophy, and more. We use this term to refer to these different fields of study for brevity purposes.

This is a *PLOS Computational Biology* Benchmarking paper.

## Introduction

With respect to race, ethnicity, gender, disability status, and LGBTQ+ (lesbian, gay, bisexual, transgender, queer, and more) identities, the science, technology, engineering, and mathematics (STEM) disciplines are persistently and significantly less diverse than the general population [1–3]. Underrepresented groups in the sciences face daily challenges such as damaging stereotypes, hostile educational environments, and discrimination, all of which are exacerbated by systemic issues in higher education [4–8]. Dismantling oppressive structures and creating new ones that value and uphold diversity, equity, inclusion, and justice as foundational principles require continuous learning, discussion, and action from all community members, especially institutional leadership.

There have been many suggestions on how to make academia more diverse, many of which are included in the other 10 rule articles [9–11]. There was a rapid growth of Diversity, Equity, and Inclusion (DEI) job opportunities and activities following the murder of Breonna Taylor, George Floyd, and many other Black Americans. The sudden interest by non-DEI professionals in performing DEI work highlighted a few things—(1) despite being undervalued or viewed as supplementary by many, DEI work also requires highly specialized field knowledge (history, sociology, philosophy, and more) and is considered a field of scholarship; and (2) good intentions are insufficient to negate the potential for harm; and (3) STEM programs, especially graduate level, need to promote interdisciplinary study of social science and humanities fields to understand how basic science research can impact society at large [12,13]. There are numerous examples where scientists have caused significant harm to others to advance science and society (their “best intentions”). We hope that our recommendations serve as the beginning of your educational journey into understanding how DEI influences all aspects of academia, including STEM research, and taking action to create a better educational environment for future generations of marginalized people.

While action is essential, inappropriate action can cause more harm than good. Self-education on these topics is a vital starting point. There is robust literature on theories and interventions about improving diversity and inclusion that we can all learn from. We recommend engaging with DEIJ literature through a discussion-based journal club to foster a sense of community and facilitate productive discussions. A well-structured journal club provides a valuable platform for learning about DEIJ theories and developing effective strategies to address equity gaps in your environment. Developing a deeper understanding of DEIJ empowers participants to advocate for and apply inclusive frameworks to their teaching pedagogy, departmental practices, and other institutional contexts.

We are careful not to understate the care required when creating a DEIJ journal club. Conversations about diversity, equity, inclusion, and justice are often difficult, emotional, and uncomfortable. This poses great difficulty in ensuring participants feel safe within the discussion space. Individuals participating in discussions should differentiate between situations threatening their psychological safety [14] and those making them feel uncomfortable. While threats to psychological safety are unacceptable, discomfort may be an expected part of the process. Further, we acknowledge that creating a DEIJ journal club may become the responsibility of marginalized people within an institution, contributing to the minority tax that plagues academia [15]. To mitigate this tax, we encourage individuals from privileged backgrounds to participate in the creation and stewardship of the journal club. Finally, we present these recommendations as, exactly that, *recommendations*. We encourage and welcome conversations about best practices for increasing diversity, equity, inclusion, and justice in academia (and beyond).

The recommendations we provide are informed by the principles of restorative justice [16]. Restorative justice practices emphasize building and repairing community relationships, emphasizing understanding and resolution over punishment when harm occurs. To establish trust among participants, great care is required to create an environment that acknowledges and respects the sensitive and personal nature of the topics discussed, some of which reflect participants’ lived experiences. Our recommendations are aimed at building community cohesion through education and mutual understanding. Crafted with intention and care, the journal club can serve as the foundation for building a safe and inclusive community within your lab or department.

Herein, we offer 10 recommendations for creating a DEIJ journal club. These suggestions are based on our experience establishing and running a DEIJ journal club in the Fraser Lab (https://fraserlab.com/) at the University of California, San Francisco (UCSF). Our journal club meets monthly, with a designated discussion leader who selects the paper. The discussion leader summarizes the paper’s findings, guides a robust conversation, and synthesizes relevant points and action items in a blog post (https://fraserlab.com/tags/deij_jc/). While not all of the recommendations may seem applicable to your institution, we are confident that these guidelines offer a solid foundation for those eager to have profound conversations about DEIJ.

### Recommendation 1: Decide on the scope of your journal club

There is vast scholarship in history, sociology, bioethics, science and technology studies, discipline-based education research, and more. Defining the scope of your journal club enables the group to identify relevant articles, foster meaningful discussions, and brainstorm solutions for ongoing DEIJ issues. To help define the focus of your journal club, ask yourself the following questions:

- Do you want to read about DEIJ research within the context of academia, industry, or public policy?
- Do you want to focus on DEIJ-research conducted in a specific field, i.e., life sciences, social sciences, mathematics, engineering, etc?
- Do you want to focus on a specific identity or experience, i.e., gender, race, disability, etc?
- Do you want to focus on a specific aspect of academia, i.e., recruitment, retention, or sense of belonging?

These questions are meant to inspire your journal club participants to reflect on what issues are important to them and what areas of knowledge they want to explore more deeply. Given the breadth of topics available, we created a repository of DEIJ articles (https://bit.ly/DEIJ_articles). This repository allows us to share articles with our colleagues and build a network of research scholarship that can be discussed in future journal club meetings. We have created an article submission form for others to contribute to this repository (https://bit.ly/DEIJ_submit). It is important to remember that while defining the scope helps promote productive conversations, you can always change the scope in response to current events and the group’s evolving interests.

### Recommendation 2: Establish discussion expectations, boundaries, and conflict resolution protocols

Discussions about how systems of oppression negatively influence academia (and society at large) can sometimes result in discomfort and tension among participants. For healthy and productive discussions, all participants must feel safe to share their thoughts and/or personal experiences. Defining boundaries as a group helps create a space for difficult but important conversations. In addition to setting boundaries, defining clear expectations of participants is important for minimizing miscommunication and the potential for harm. Based on established restorative justice principles, here are some boundaries and expectations we established in our own journal club:

- Be prepared—Participants are expected to have read the article, ensuring an informed discussion on its content.
- Respect others—This includes listening to each other’s perspectives, avoiding interrupting or talking over one another, and refraining from using offensive or inflammatory language.
- Reflect before responding—If you feel yourself getting defensive during a discussion, take a moment to reflect on what you’re feeling. Reflect if you’re feeling discomfort or a threat to your psychological safety, and respond accordingly.
- Maintain confidentiality—It is imperative for all participants of the journal club to respect the privacy of personal experiences shared during the discussions and refrain from sharing those stories outside of the group without obtaining consent from the relevant participants. The discussion summary will capture the essence of these stories while omitting any identifying information.

Even with defined expectations and boundaries, it is still possible that conflict or harm between participants may arise during a discussion. While we do not expect all participants to be expertly trained in restorative justice practices, we believe it is important for participants to be able to engage in conflict resolution in this space. Some elements of a restorative mindset [17] that promote a safe discussion space include:

1. Communal Mindset: We are dedicated to the community’s future, understanding that it’s a blend of diverse individuals united for a shared purpose.
2. Justice Perspective: “We do justice with people and not to them.”—Fania Davis. Justice is for all parties involved, including victims, communities, and wrongdoers.
3. Centering Solutions: We address the needs of those affected, questioning what’s essential for the right relationships.
4. Collaborative Approach: We embrace teamwork, emphasizing a joint effort in problem-solving.
5. Restoration Focus: We prioritize respect, dignity, and care, aiming to restore both the harmed and the perpetrator, as well as the broader community.

If a discussion becomes contentious or unsafe, the discussion leader should attempt to facilitate conflict resolution, beginning with reiterating the community expectations and restorative mindset principles. If resolving the conflict does not seem feasible, the discussion leader should end the current session and seek support from external partners trained in conflict resolution and/or restorative justice practices. There should not be any additional journal club discussions until the harm caused has been addressed and steps forward for healing the group have been identified.

### Recommendation 3: Rotate discussion leaders to promote diverse conversations and distribute responsibilities

We encourage rotating discussion leaders to foster active participation and distribute responsibilities among participants. For each session, the discussion leader selects an article (see **Recommendation #4**), guides the conversation, and ensures respectful engagement. By rotating leaders, we not only share the responsibility of organizing and leading the journal club but also allow everyone the opportunity to discuss a topic that they find interesting or relevant.

The discussion leader’s first responsibility is to pick an article (see **Recommendations #1** and **#4**) and read any relevant background literature. We suggest reading related works, beginning with those cited in the article and works that cite the article (if available). Once the discussion leader has selected the article, they need to notify the participants with sufficient time to read the article and any additional recommended materials.

The second responsibility of the discussion leader is to prepare for the discussion. Preparation is instrumental to fostering an engaging and productive conversation and swiftly navigating differences in opinion. The discussion leader should have a thorough understanding of the article, its thesis, and the frameworks used by the authors. Given the nature of these discussions, discussion leaders need to reflect on their personal identities and biases to understand how these factors influence their interpretation and discussion of the article [18]. An example of self-assessment of one’s positionality: a cisgender male discussion leader presenting an article about the negative impacts of gender bias on the career advancement of cisgender women, non-binary, and transgender people in STEM. While the discussion leader may have a theoretical understanding of gender bias, he has not experienced gender bias directly and, therefore, lacks a more nuanced understanding of gender bias. Acknowledging the lived experiences of participants is important for informing discussions and helps guide the development of solutions to the problems discussed.

Once the discussion leader has reviewed the relevant materials and performed this self-reflexive exercise, they should develop questions to help guide the discussion. When writing these questions, the discussion leader should reflect on their personal identities and how their experiences may influence the questions they ask. In addition, the leader should be mindful to avoid simplistic assumptions or oversights that could trigger trauma or cause harm.

The third responsibility of the discussion leader is to lead the discussion! In our experience, we have found that discussion-based journal clubs allow people to express their opinions, explore the limits of their knowledge and comfort, and imagine novel solutions*. Discussion leaders should be aware of the power dynamics within the group, whether based on personal identities or professional roles. Depending on the topic, creating discussion groups by career stage (graduate students only, faculty only, etc.) may allow for more candid conversations. However, there is value in discussions across career stages, and these opportunities should be offered as long as a safe and respectful environment is guaranteed.

At the beginning of each journal club, the discussion leader should reiterate the expectations, provide a brief overview of the article, and explain why they selected this article. The discussion leader should allocate time at the end of the session for participants to develop action items and key take-aways. While this is not required, we have found it useful to have the discussion leader write a summarizing blog post for each journal club session.

*Authors’ note: There is no right way to operate a “discussion-based” journal club, so we encourage you to discuss with your participants what format and tools will help everyone engage with the material. For some, this means beginning with a summary presentation by the discussion leader prior to an open dialogue. For others, the entire session may be open dialogue. Do whatever feels right for your group.

### Recommendation 4: Select an article for discussion

The core of a journal club is, spoiler alert, the articles. Discussion leaders should choose an article they feel comfortable discussing in-depth (see **Recommendation #3**). Leaders can build that comfort by reviewing supplemental materials or talking with subject experts to ensure that they are prepared to guide a fruitful discussion. Thoughtful article selection and discussion preparation will help cultivate productive discussions and encourage participants to identify connections between the article and their environment. In addition to the article topic (see **Recommendation #1**), you should also consider the type of article, article length, and the academic background of your journal club participants. Given that most articles will be outside of your group’s area of study, consider providing optional supplemental readings to provide context for your main discussion article.

Scholarly articles come in various types, such as theoretical works, observational or interventional case studies, and commentaries. Using diverse types of articles provides complementary perspectives and provides participants with a deeper understanding of each article selected for discussion. Theoretical articles can provide a comprehensive overview but are often more dense and require substantial background knowledge. Observational or interventional studies focus on practical applications of theoretical frameworks and are most similar to life sciences research articles. Commentary or opinion pieces typically provide more accessible insight into a complex topic, often incorporating anecdotal and/or personal experiences to support the author’s argument.

However, due to their content and writing style, different article types will require varying amounts of background knowledge and supplementary reading by participants. It is important to account for this and provide adequate time and resources for participants to prepare for discussion. Pairing different types of articles, such as a theory article with a commentary piece, provides the reader with primary literature and a more accessible summary of that literature. Likewise, recommending a theoretical or commentary piece in addition to the main interventional study article provides additional context to help the reader understand the study’s design and data analysis methods.

### Recommendation 5: Continue the conversation outside of your regularly scheduled sessions

One goal of forming a DEIJ journal club is to continue to discuss DEIJ beyond your scheduled sessions, slowly (or rapidly) integrating these topics into lab or department discussions. By talking about DEIJ outside of your journal club, you begin to signal to others that these discussions are normal and appropriate for academic spaces.

One way you can begin to incorporate DEIJ into your daily life is by reflecting on the articles you’ve read and considering how you can apply any recommendations or findings from those articles to your environment. For example, if you recently read an article about how bias can affect interview outcomes during graduate recruitment or hiring, and the authors provide tools on how to combat these biases—talk to your supervisor about implementing these tools in your recruitment and hiring process.

Another way to incorporate DEIJ into your daily life is by asking your faculty mentor or department chair to dedicate space (Slack channel, bulletin board, ect.) or time (5 to 10 min) in recurring research settings for people to share about DEIJ news and updates (https://fraserlab.com/2020/06/26/Minute-For-Diversity/).

### Recommendation 6: Actions speak louder than words

Educating oneself on DEIJ is necessary but not sufficient. We must transform what we’ve learned into action to make a difference. We hope that by creating a space where like-minded individuals can connect, interact, and strategize, you will cultivate a community of scientists who are passionate about advancing equity and inclusion and empowered to take action.

Discussion sessions should allocate time to brainstorming action items. These items can range from small, personal changes to more substantial local or institutional changes. By closing the journal club session with a call to action, the group must synthesize the article’s findings and theoretical frameworks and identify aspects of their environment that are susceptible to positive change.

Sharing is caring—consider sharing your discussion summaries with your colleagues. We relied on blog posts and social media (R.I.P. to Science Twitter) to share our summaries with the broader scientific community. When drafting your discussion summary, refer to specific data in the article to support your proposed action items. Inquire your faculty mentor and/or department chair about the funding and institutional resources available to facilitate the implementation of these action items. If you, dear reader, hold a leadership role in your lab, department, or institution, use that opportunity to advocate for implementing these action items.

Even though you may just be starting your DEIJ journey, we encourage you to think big about your potential impact on your institution. Suppose you can get your department to value and promote DEIJ activities. In that case, this will have positive implications on departmental culture, ranging from incorporation of DEIJ curriculum in graduate education to increased recruitment and retention of trainees and faculty from marginalized backgrounds. There is immense value in understanding how your work at the bench translates into the real world (e.g., the development and distribution of the COVID-19 vaccine and the resultant public response (and backlash) against this novel therapeutic). Any positive change you enact may be the snowball that causes an avalanche.

### Recommendation 7: Keep your journal club sustainable

Running a successful journal club requires consistent effort and resources. First and foremost, creating a welcoming and inclusive environment is an ongoing process. It requires centering restorative practice to encourage engagement from all participants, especially those from marginalized backgrounds.

Running a journal club also demands significant time and resources, which can be challenging when all participants are volunteers. Here are some considerations to help reduce the burden of organizing a journal club and keep this activity sustainable:

- Designate someone in your lab or department as a journal club coordinator as part of their formal job description. Their role will be to help with scheduling, advertising, ordering food, and other administrative tasks.
- Consider the frequency and length of sessions. While meetings should be frequent enough to facilitate community building, participants should have enough time to engage with the literature and reflect on it before meeting as a group.
- Advertise your journal club broadly to attract new participants. Talk with leadership to ensure that departmental seminars and other large meetings are not scheduled in conflict with your journal club.
- Solicit your lab or departmental leadership for financial and administrative support. Institutional support can help the journal club facilitator with room booking, advertising, and reminder emails, as well as provide funding for material costs, such as printing or presentation tools and refreshments for attendees—who doesn’t love a free lunch?

### Recommendation 8: Evaluate the impact of your journal club

Periodically evaluating the impact of your journal club will allow you to continue facilitating meaningful discussions and promoting change in your community. First, define what a successful journal club looks like. In the Fraser Lab, we defined success as (1) the percentage of lab participation; (2) the number of discussions conducted annually; and (3) the effective implementation of new lab policies. Success will look different depending on your group’s goals and objectives, but it should be specifically defined to guide your efforts. Some metrics of success you may consider include:

- Number of participants in the journal club.
- Presenting and discussing journal club topics in larger group settings, such as lab or department-wide meetings.
- Implementation of new inclusive lab policies.
- Number of advocacy meetings with department or university administration.
- Hosting talks by journal club article authors.
- Inviting social scientists or historians as guest speakers to your journal club.
- Assisting another lab or department in starting a DEIJ journal club.

Your group should periodically revisit its definition of success and update it as necessary. It is important to note that not all discussions will lead to tangible outcomes, and the absence of such outcomes should not be considered a failure.

On an annual basis, solicit participant feedback using an anonymous numerical evaluation form where respondents can provide honest feedback. Feedback should be reviewed and summarized by someone outside of your lab or department to ensure anonymity and candor. Some suggested questions that can be answered using a Strongly Disagree to Strongly Agree scale include:

1. I feel my opinions and thoughts are valued by other journal club participants.
2. I think that people come to journal club equally prepared.
3. I believe we abide by our community guidelines.
4. I enjoy the types of articles we read.

Once you have gathered feedback, review the data as a group and update your journal club’s expectations, boundaries, and conflict resolution protocols as appropriate (see **Recommendation #2**).

### Recommendation 9: Expand your network

Your experience and understanding of a lab or department’s culture, combined with the expertise of social scientist scholars, can create a formidable synergy. We encourage you to invite scholars outside of your discipline to attend a journal club or to speak on a specific topic. Solicit funding from your graduate program, lab PI, and/or department chair so you can compensate the speaker for their time. Social scientists and humanities scholars can provide field-specific insights into the articles you are discussing, offering a new perspective to consider in future discussions.

### Recommendation 10: Help others start their own journal club!

If your journal club becomes very popular (WOO! You created something people want to be a part of!), we recommend creating smaller discussion groups (approximately 6 to 8 people) to facilitate productive discussions where all participants are able to contribute. You can always convene these smaller groups together and have someone report back from each group on what they discussed.

If someone from another lab or department wants to start their own journal club, invite them to attend yours so they can develop ideas about how to structure their own. Meet with them to discuss what has worked, what hasn’t, and what you are considering changing in the future. Oh, and tell them to read this article.

If you are a faculty member involved in a graduate program, consider the impact that a DEIJ journal club might have if incorporated into the curriculum as a course. The creation of numerous DEIJ-focused courses at UCSF, including GRAD 202: Racism in Science and Medicine (https://graduate.ucsf.edu/grad-202-racism-in-science), GRAD 210: Justice, Equity, Diversity, and Inclusion Academic Leadership (https://graduate.ucsf.edu/grad-210-dei-leadership), GRAD 219: Special Topics in Racism and Social Justice in Science, has provided students with the opportunity to explore humanities and social science literature and consider how science has been used as a tool for oppression. By providing educational opportunities that bridge one’s research in the lab to the outside world around them, you reinforce that scientific research does not exist in a vacuum and that scientists need to have a sense of social responsibility beyond their immediate scientific community.

## Conclusion

Dear reader, we’ve reached the end of our time together. We hope you’ve learned something about starting a DEIJ journal club and feel empowered to start your own. Facilitating discussions about DEIJ in academia is just the beginning. Advancing DEIJ requires developing equity-mindedness, uncovering systemic inequities, and engaging in educational change-making strategies. We hope that creating a DEIJ journal club will unite people interested in this work and nucleate a coalition of DEIJ leaders. Email us to let us know how your journal club goes! We encourage readers to share their experiences via blog posts or social media and join the growing community of social justice-oriented scientists. By expanding our networks and sharing our experiences, we begin to create a more inclusive and equitable academic environment for all marginalized people.
